# The estimation of long and short term survival time and associated factors of HIV patients using mixture cure rate models

**DOI:** 10.1186/s12874-023-01949-x

**Published:** 2023-05-22

**Authors:** Khadijeh Najafi Ghobadi, Ghodratollah Roshanaei, Jalal Poorolajal, Ebrahim Shakiba, Kaivan KHassi, Hossein Mahjub

**Affiliations:** 1 Department of Biostatistics, School of Public Health, Hamadan University of Medical Sciences, Hamadan, Iran; 2 Modeling of Noncommunicable Diseases Research Center, Hamadan University of Medical Sciences, Hamadan, Iran; 3 Research Center for Health Sciences, Hamadan University of Medical Sciences, Hamadan, Iran; 4 Department of Epidemiology, School of Public Health, Hamadan University of Medical Sciences, Hamadan, Iran; 5 Behavioral Disease Research Center, Kermanshah University of Medical Sciences, Kermanshah, Iran; 6 Health Department, Kermanshah University of Medical Sciences, Kermanshah, Iran; 7 Department of Biostatistics, School of Public Health and Research Center for Health Sciences, Hamadan University of Medical Sciences, Hamadan, Iran

**Keywords:** HIV, Mixture cure model, Mixture cure frailty model, Cure fraction, Survival analysis

## Abstract

**Background:**

HIV is one of the deadliest epidemics and one of the most critical global public health issues. Some are susceptible to die among people living with HIV and some survive longer. The aim of the present study is to use mixture cure models to estimate factors affecting short- and long-term survival of HIV patients.

**Methods:**

The total sample size was 2170 HIV-infected people referred to the disease counseling centers in Kermanshah Province, in the west of Iran, from 1998 to 2019. A Semiparametric PH mixture cure model and a mixture cure frailty model were fitted to the data. Also, a comparison between these two models was performed.

**Results:**

Based on the results of the mixture cure frailty model, antiretroviral therapy, tuberculosis infection, history of imprisonment, and mode of HIV transmission influenced short-term survival time (*p*-value < 0.05). On the other hand, prison history, antiretroviral therapy, mode of HIV transmission, age, marital status, gender, and education were significantly associated with long-term survival (*p*-value < 0.05). The concordance criteria (K-index) value for the mixture cure frailty model was 0.65 whereas for the semiparametric PH mixture cure model was 0.62.

**Conclusion:**

This study showed that the frailty mixture cure models is more suitable in the situation where the studied population consisted of two groups, susceptible and non-susceptible to the event of death. The people with a prison history, who received ART treatment, and contracted HIV through injection drug users survive longer. Health professionals should pay more attention to these findings in HIV prevention and treatment.

**Supplementary Information:**

The online version contains supplementary material available at 10.1186/s12874-023-01949-x.

## Introduction

Human immunodeficiency virus (HIV) is still one of the most critical public health issues globally, as almost 33 million people have died from the virus so far. By taking HIV medicine, antiretroviral therapy (ART), infected people can live a long and healthy life. Nevertheless, without treatment a person with HIV is more likely to develop a severe condition called acquired immunodeficiency syndrome (AIDS). If left untreated, the life expectancy at the final stage of AIDS is about three years [1].

At the end of 2019, about 38 million people were living with HIV, of whom 81% knew their status. Due to the gap in HIV services, 690,000 people died at the end of 2019 due to HIV-related causes, and 1.7 million people have recently been infected with the virus [1–3]. Iran has been one of the active countries in fighting against HIV/AIDS in the Middle East for decades [4]. However, despite the efforts made, there are 59,000 (33,000–130,000) people living with HIV in this country, and 4,100 (1200–12,000) people have recently been infected with the virus, of which about 2,500 (1200–5600) have died of AIDS [5].

In the contemporary world, extensive research is being done to increase the lifetime of patients with a variety of diseases, including deadly diseases such as cancer and HIV / AIDS. As a result, many patients are expected to be cured permanently. They are long-term survivors and do not experience so-called death. These people are called cured patients [6]. In such a situation, where the follow-up period is long and a proportion of patients do not experience the event of interest (non-susceptible), the rate of censorship increases, and as a result, the estimated survival rate of conventional methods such as the Cox model is overestimated because Cox model regards all the patients as susceptible [7, 8].

When a non-negligible proportion of patients long survive (non-susceptible), mixture cure models describe to survival process more appropriately [8]. For several reasons, mixture cure models can be an excellent alternative to the conventional Cox proportional hazard models for high-censorship data. First, proportional hazards may not be assumed when the survival curves in their sequence are flat (horizontal line). Second, survival curves with a long horizontal line in the sequence may indicate heterogeneity in patients’ populations, which can be helpful for explicitly describing mixture cure models [8]. Mixture cure models make it possible to estimate the proportion of long-term survivors and investigate the effect of covariates on short- and long-term survival time [9]. In several studies, the cured models have been theoretically developed and in the proposed models, HIV data have been used [10–13]. Varshney et al.’s study estimated the proportion of cured people living with HIV/AIDS treated with ART using Bayesian methods [8].

Price and Manatunga have pointed out that in medical and epidemiological studies, there may be some unobserved covariates between individuals that observed variables in survival analysis cannot explain. If these unobserved heterogeneities are not accounted for, the results will be biased [14]. In mixture cure models, random effects can be used to compensate for the lack of information on other important covariates and controlling some individual characteristics that effect on survival time. Such models are referred to as mixture cure frailty models [15].

Many studies have been conducted on the mixture cure frailty models [16–18]. For example, Calsavara introduced the long-term frailty model using a non-proportional risk model and applied this model to melanoma datasets [19]. Other studies have used the mixture cure model for competing risks [20–22]. Ghavami used the parametric mixture cure gamma frailty model and vertical modeling of competing risks for breast cancer data in her study [23].

To our knowledge, no study in Iran has performed the factors affecting the short- and long-term survival time of HIV patients using mixture cure models. For this reason, in the present work, to estimate the probability of long-term survival of each HIV-infected individual and the factors affecting the mortality of HIV-infected individuals, a semi-parametric mixture cure model was applied. Also, to compensate for the lack of information on other important covariates, a mixture frailty model was used.

## Materials and methods

### Data

In this study, the data of 2170 HIV-infected people referring to the Kermanshah Province Disease Counseling Centers from 1998 to 2019 were examined. The study was a retrospective cohort study. The information on baseline demographic and clinical variables, including diagnosis age, gender, history of imprisonment, history of addiction, education, marital status, occupation status, ART, modes of HIV transmission, and tuberculosis infection was collected from medical records. We included those people who were diagnosed as HIV + /ADIS based on two positive Elisa tests and one positive Western blot test or a rapid HIV test and two positive Elisa tests (that one the fourth generation of HIV ELISA test), which are used now simultaneously irrespective of nationality, age, and gender. We had no exclusion criteria. The outcome of the interest was time to diagnosis until death (survival time). All the patients who were alive at the end of the study were censored for death.

### Semiparametric PH mixture cure model

The mixture cure model is a specific type of survival model. In these models, the studied population is assumed to be a mixture of uncured, susceptible, individuals who may experience the event of interest and cured individuals, non-susceptible, who will never experience the event. The advantage of using this model over conventional survival models is that the estimated probability of long-term survival and its effective factors can also be analyzed in this model simultaneously.

Let *T* denote the failure time of interest, $1 - \pi ({\mathbf{Z}})$ be the probability of a patient.being cured and $S(t|{\mathbf{X}})$ be the survival probability of an uncuredpatient. Where, ***X*** and ***Z*** are observed values of two covariate vectors that may affect the survival function and probability of the uncured, respectively. Boag suggests a mixture cure model $S_{pop} (t|{\mathbf{X}},{\mathbf{Z}})$, in which $S(t|{\mathbf{Z}}) = 1$ for the cured group in the population [24]. A mixture function was used as follows:

$$S_{pop} (t|{\mathbf{X}},{\mathbf{Z}}) = \pi ({\mathbf{Z}})S(t|{\mathbf{X}}) + 1 - \pi ({\mathbf{Z}})$$

Usually, $\pi ({\mathbf{Z}})$ is referred to as “incidence" and $S(t|{\mathbf{X}})$ is “latency". The proportional hazard (PH) model is used to model the latency part, the cure model is called the PH mixture.cure model. A logit link function $\pi ({\mathbf{Z}}) = \frac{{\exp ({\mathbf{bZ}})}}{{1 + \exp ({\mathbf{bZ}})}}$, where ***b*** is a vector of unknown parameters, is used to model the effects of ***Z*** covariate vector. Proportional hazard models describe the event time distributions given to uncured patients. The proportional hazards model is defined as $h(t) = h_{0} (t)\exp ({\mathbf{\beta X}})$, where $h_{0} (t)$ is the arbitrary baseline hazard at time *t*, where ${{\varvec{\upbeta}}}$ is the regression coefficient of the covariate vector **X**.

The EM algorithm was used to estimate the unknown parameters in the mixture cure model [20]. To estimate the mixture cure model parameters, the follow-up time should be sufficiently long and the sample size should be large [25].

The Kaplan Meier curve and the Maller and Zhou test were used to test the hypothesis of curing (presence of patients with long-term survival time). If there were cured patients, the Kaplan–Meier curve becomes horizontal before reaching zero [26, 27].

### Mixture cure frailty model

Suppose there is a latent binary variable $v_{i}$, $v_{i} = 0$ if subject *i* is cured, and $v_{i} = 1$ if subject *i* is not cured. We denote the probability of not being cured by $\pi_{i}$ and define it as follows:

$$\pi_{i} = p(v_{i} = 1|{\mathbf{Z}}) = g({\mathbf{Z\theta }})$$

where g (·) is a logit link function, ${\mathbf{Z}}$ is a vector of covariates, and ${{\varvec{\uptheta}}}$ is the regression coefficient vector. If person *i* is uncured $v_{i} = 1$, the hazard function at time *t* is defined as follows.

$$h_{i} (t|w_{i} ,{\mathbf{X}},\nu_{i} = 1) = w_{i} h_{0} (t)\exp ({\mathbf{X}}^{T} {{\varvec{\upbeta}}})$$

where ${{\varvec{\upbeta}}}$ is the regression coefficient of the covariate ***X***, $w_{i}$ is the subject-specific frailty and follows a Gamma distribution, $\Gamma (\Psi^{ - 1} ,\Psi )$, where $\Psi$ is the variance of the frailty distribution. The probability density function for $w_{i}$ is

$$f(w_{i} |v_{i} = 1) = w_{i}^{{\frac{1}{\Psi } - 1}} \exp ( - \frac{{w_{i} }}{\Psi })/\Gamma (1/\Psi )\Psi^{{\frac{1}{\Psi }}}{.}$$

In addition, $h_{0} (t)$ is the arbitrary baseline hazard function. Let $\Omega_{i} = \{ {\mathbf{X}},{\mathbf{Z}},t_{i} ,\delta_{i} :i = 1,...,n\}$ denote the observed data in subject *i.* The complete data likelihood for subject *i* can be written as:

$$L_{i,c} = (1 - \pi_{i} )^{{1 - \nu_{i} }} (\pi_{i} G_{i} )^{{\nu_{i} }}$$

where

$$G_{i} = w_{i}^{{\frac{1}{\Psi } - 1}} \exp ( - \frac{{w_{i} }}{\Psi })/\Gamma (1/\Psi )\Psi^{{\frac{1}{\Psi }}} ([w_{i} h_{0} (t)\exp ({\mathbf{X}}^{T} {{\varvec{\upbeta}}})]^{{\delta_{i} }} \exp \{ - w_{i} H_{i} (t)\} )$$

and

$$H_{i} (t) = \int\limits_{0}^{t} {h_{0} (s)} \exp \{ {\mathbf{X}}^{T} (s)\beta \} ds.$$

The complete data likelihood of all the study subjects is $L_{c} = \Pi_{i = 1}^{n} L_{i,c}$. It was used to estimate the unknown parameters of the EM algorithm for this model [28].

The probability of cure for each person was calculated using the formula $1-\widehat{p}=1-(\mathrm{exp}({\varvec{Z}}\widehat{{\varvec{\theta}}})/(1+\mathrm{exp}(Z\widehat{\theta })))$, with the coefficients resulting from the fitted model and putting each person's characteristics into the formula.

The missing data were imputed using the multiple regression method [29, 30]. Also, in order to check collinearity between the variables, the variance inflation factor (VIF) was used [31]. We used concordance criteria (K-index) to evaluate the goodness of fit of the two models.

In this study, R 4.1. software (the smcure package) and STATA 16.0 (the strmcure command) were used for analysis [28, 32]. We used concordance criteria in the eva_cure package to check the K-index [33]. The significance level was considered 0.05.

## Results

The mean and median follow-up times were 173.75 and 119.13 months, respectively. About 50% of the patients had more than 33 years old. Of these, 57.5% were censored. Among patients, 83.5% were men, 52.1% were married, and 58.0% were unemployed. In addition, 97.1% had a low level of education, and 62.6% had a history of imprisonment. The frequency of drug users was 74.2%. Also, 54.3% of people did not receive ART. About 67.1% were infected through HIV-injecting drug users. The number of infected with tuberculosis was 67.9% were. Further information is provided in Table 1.

**Table 1:** Descriptive analysis of HIV-infected (*n* = 2170)

Variables	Status patients	
	**Total N(%)**	**Dead N(%)**
**Gender**		
Male	1811 (83.5)	879 (95.2)
Female	359 (16.5)	44 (4.8)
**Age**		
< = 33	1082 (49.9)	465 (50.4)
> 33	1088 (50.1)	458 (49.6)
**Marital Status**		
Single	1039 (47.9)	548 (59.4)
Married	1131 (52.1)	375 (40.6)
**Educational Level**		
Low (illiterate to high school)	2106 (97.1)	913(98.9)
High (academic)	64 (2.9)	10 (1.1)
**imprisonment**		
No	811 (37.4)	329 (35.6)
Yes	1359 (62.6)	594 (64.4)
**Drug Abuse**		
No	560 (25.8)	158 (17.1)
Yes	1610 (74.2)	765 (82.9)
**Antiretroviral Therapy**		
No	1178 (54.3)	784 (84.9)
Yes	992 (45.7)	139 (15.1)
**Occupational status**		
Unemployed	1259 (58.0)	484 (52.4)
Employed	911 (42.0)	439 (47.6)
**Modes of HIV transmission**		
Other	714 (32.9)	123 (13.3)
Injecting drug users	1456 (67.1)	800 (86.7)
**Tuberculosis infection**		
No	697 (32.1)	425 (46.0)
Yes	1473 (67.9)	498 (54.0)

**Fig. 1:**
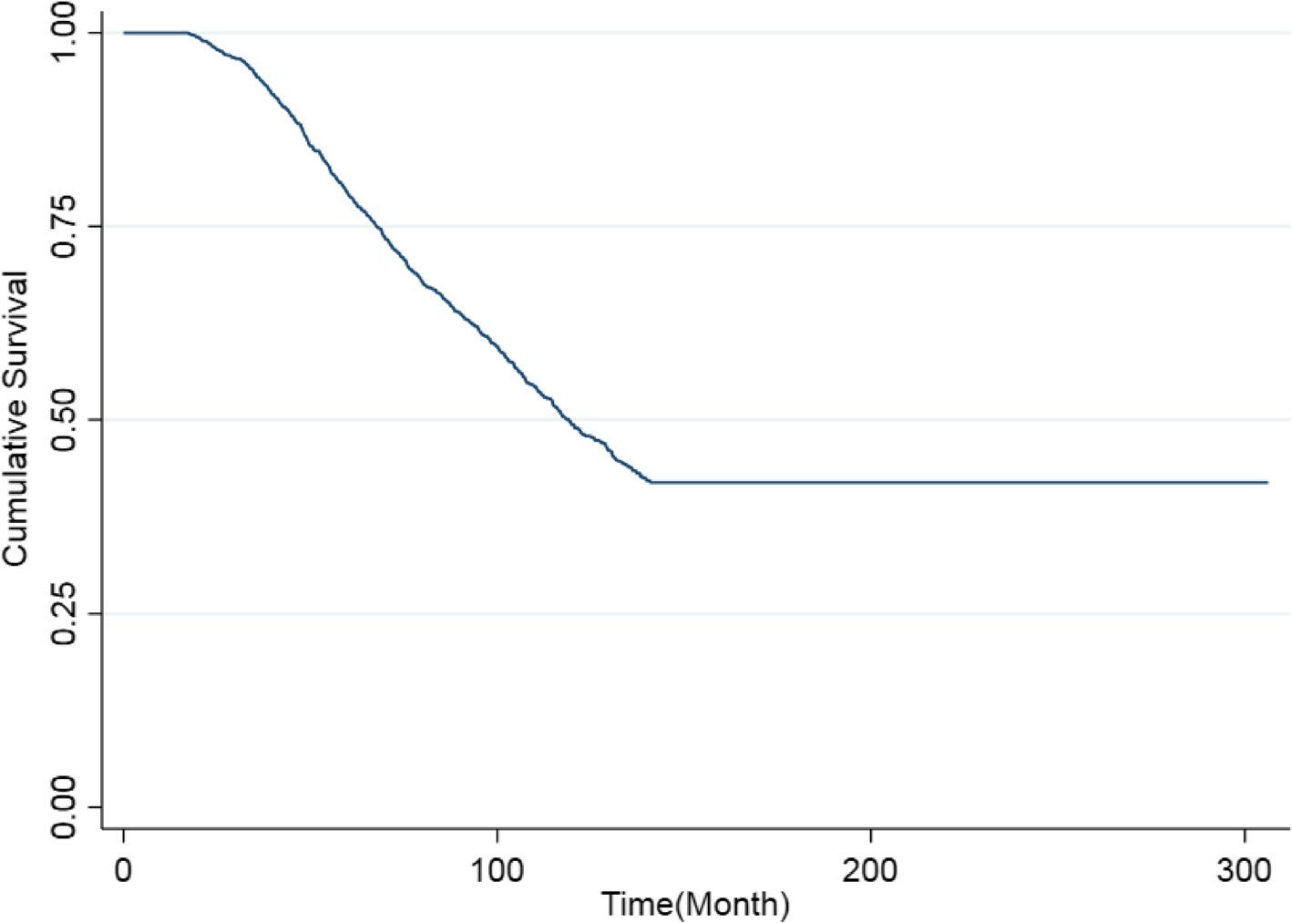
Kaplan -Meier curve for the whole HIV data

As shown in Fig. 1, the Kaplan -Meier curve becomes flat above zero. The Kaplan–Meier plot shows a clear plateau of about 141 months for death which could justify the use of the cure models. Also, the results of Maller and Zhou's test show that there are patients with long-term survival in the present study. Table 2 shows the results of the semi-parametric mixture cure model and mixture cure frailty model in the presence of covariates. The K-index value of the frailty model was better than that of the mixture cure model and was 0.65 against 0.62. The results of the mixture cure frailty model showed that variables of ART, tuberculosis infection, history of imprisonment and mode of HIV transmission affected death (*p*-value < 0.05). The variables of age, ART, education, gender, marital status, history of imprisonment and mode of HIV transmission are significant in the probability of being cured of death (*p*-value < 0.05). The results of this model also show that the frailty component is significant (*p*-value = 0.001). It means that there are some other important variables and some individual characteristics that were not considered in the present study.

**Table 2:** Results of semiparametric PH mixture cure model and mixture cure frailty model fitting on HIV patients

semiparametric PH mixture cure model				mixture cure frailty model		
**Cure probability model (Incidence model)**						
**Variable**	**Coefficient**	**SE**	**OR(95% CI)**	**Coefficient**	**SE**	**OR(95% CI)**
Intercept	0.518	0.354	1.679(0.839,3.360)	0.544	0.305	1.723(0.948, 3.132)
Age(> 33/ < = 33)	0.509	0.145	1.664(1.252,2.210)	0.498	0.141	1.645(1.248,2.169)
Gender (Male/ Female)	1.228	0.379	3.414(1.624,7.177)	1.214	0.335	3.367(1.746, 6.492)
Marital Status (Married/ Single)	-0.327	0.138	0.721(0.550,0.945)	-0.309	0.144	0.734(0.554, 0.974)
Antiretroviral therapy (Yes /No)	-2.028	0.143	0.132(0.099,0.174)	-2.035	0.159	0.131(0.096, 0.178)
Tuberculosis infection (Yes/No)	-0.147	0.183	0.863(0.603,1.236)	-0.147	0.153	0.863(0.640, 1.165)
Education (High /Low)	-1.218	0.419	0.296(0.130,0.672)	-1.241	0.498	0.289(0.109, 0.767)
Imprisonment(Yes /No)	-1.034	0.198	0.356(0.241,0.524)	-1.047	0.198	0.351(0.238, 0.517)
Drug Abuse(Yes /No)	-0.455	0.269	0.634(0.374,1.075)	-0.444	0.281	0.641(0.370, 1.113)
Occupational status (Employed/ Unemployed)	0.166	0.122	1.181(0.929,1.499)	0.167	0.138	1.182(0.902, 1.549)
Modes of HIV transmission (Injecting drug users/Other)	0.732	0.271	2.079(1.222,3.537)	0.720	0.262	2.054(1.229, 3.433)
**Failure time distribution model (Latency model)**						
**Variable**	**Coefficient**	**SE**	**HR(95% CI)**	**Coefficient**	**SE**	**HR(95% CI)**
Age(> 33/ < = 33)	0.046	0.073	1.047(0.907,1.208)	0.115	0.094	1.122(0.933, 1.349)
Gender (Male/ Female)	-0.221	0.229	0.802(0.512,1.256)	-0.227	0.293	0.770(0.449, 1.415)
Marital status (Married/ Single)	-0.024	0.084	0.976(0.828,1.151)	-0.093	0.103	0.911(0.745, 1.115)
Antiretroviral therapy (Yes /No)	-0.632	0.121	0.532(0.419,0.674)	-0.780	0.152	0.458(0.340, 0.617)
Tuberculosis infection (Yes /No)	0.252	0.073	1.287(1.115,1.485)	0.330	0.094	1.391(1.157, 1.672)
Education (High /Low)	-0.086	0.460	0.918(0.372,2.261)	-0.052	0.491	0.949(0.363, 2.485)
Imprisonment (Yes /No)	-0.355	0.088	0.701(0.590,0.833)	-0.466	0.108	0.628(0.508, 0.775)
Drug abuse (Yes /No)	-0.143	0.107	0.867(0.703,1.069)	-0.171	0.162	0.843(0.614, 1.158)
Occupational status (Employed/ Unemployed)	-0.003	0.075	0.997(0.861,1.155)	-0.027	0.096	0.973(0.806, 1.175)
Modes of HIV transmission (Injecting drug users/Other)	0.705	0.151	2.024(1.505,2.721)	0.888	0.195	2.430(1.658, 3.562)
Frailty parameter	-	-	-	0.380	0.066	(0.249, 0.510)

**Fig. 2:**
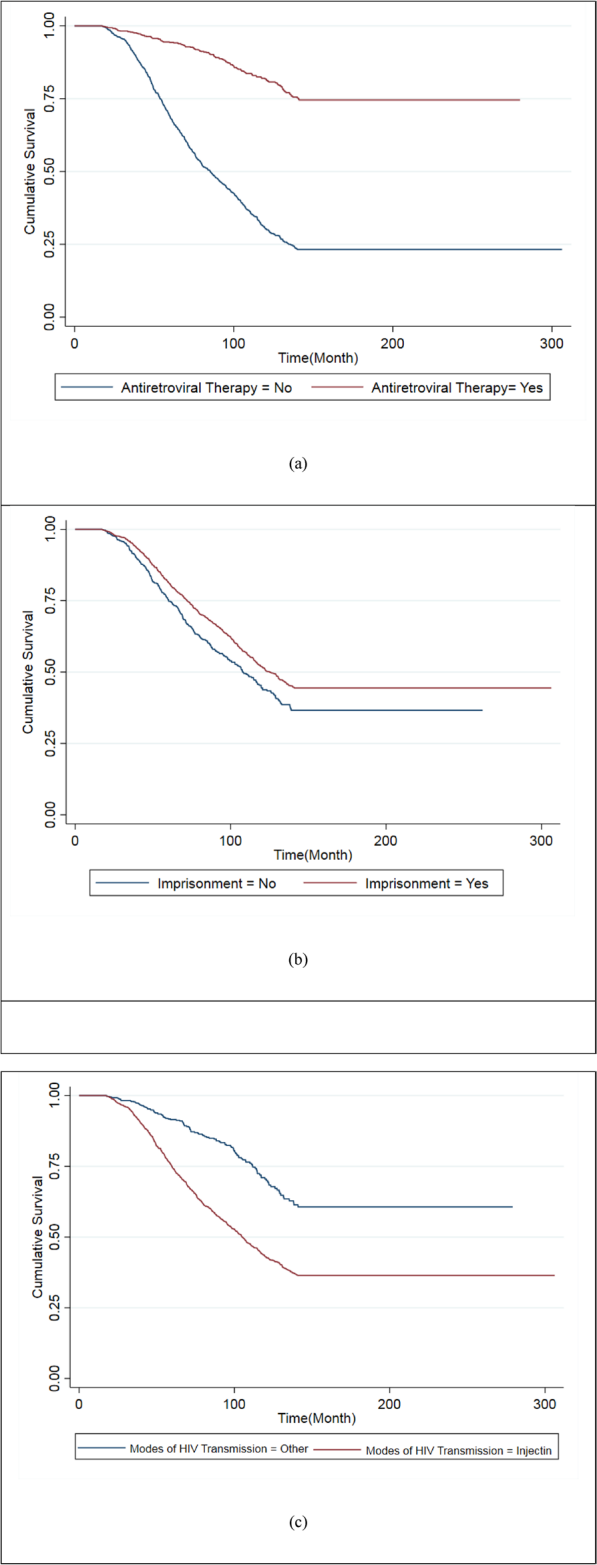
**a** Kaplan–Meier survival curves for antiretroviral therapy; **b** Kaplan–Meier survival curves for imprisonment; **c** Kaplan–Meier survival curves for modes of HIV transmission

According to the results of the mixture cure frailty model in the first part of Table 2, the cure odds in men were 3.367 times higher than in women (*P* = 0.001). On the other hand, antiretroviral therapy significantly reduced the risk of death compared to those who did not receive antiretroviral therapy (OR = 0.131; *P* = 0.001). The cure probability was significantly higher among high-educated people than among low-educated individuals (OR = 0.289; *P* = 0.013). The cure probability was much higher in prisoners than in non-prisoners (OR = 0.351; *P* = 0.001). The cure odds for persons over 33 years old is 1.645 times higher than for those under 33 years of age (OR = 1.645; *p* = 0.001). Based on the second part of Table 2, ART reduced the risk of death by almost 50% (HR = 0.458; *P* = 0.001), while coinfection with tuberculosis increased the risk by about 40% (HR = 1.391; *P* = 0.001). Prisoners were at lower risk of death than non-prisoners (HR = 0.628; *P* = 0.001). The risk of death was more than two times among injection drug users than in other modes of transmission. As shown in Fig. 2, People who received ART had higher survival than those who did not. People who had a history of imprisonment were more likely to survive longer. Also, people infected with HIV through drug injection use had higher survival than those infected through other ways.

## Discussion

Various studies have been performed in Iran and the world on the survival of HIV-infected patients. The conventional Cox method have used in some studies [34–37] and few works in some parts of the world have performed mixture cure models [8, 11]. In some investigations, the parameters estimation method was the EM algorithm, but in Davies's study [38], the stochastic EM algorithm was used to estimate the parameters. In the studies that have used the EM and stochastic EM algorithm, the goodness of fit of the expressed models is graphical methods. In some studies, the Markov Chain Monte Carlo sampling algorithm has been employed for model fitting and comparison, and the deviance information criteria (DIC) and the logarithm of pseudo-marginal likelihood (LPML) indices have been used for evaluating the goodness of fit [39–42]. However, in the present study, although the parameters estimation method was the EM algorithm, the goodness of fit index was a concordance index, which is a more common index for survival studies.

The presence of short-term and long-term survivors in the mixture cure models and promotion time cure models, may lead to a violation of the PH assumption [43]. When the PH is not established in these models, the Schoenfeld residual cannot be used to test for this feature [44]. So in this study, we checked the PH assumption for all covariates using the ln(-ln(survival)). Examining this assumption showed that the PH is not valid for the TB variable. Some authors have pointed out that the mixture cure model can be used if there is evidence of a cure fraction in the study, even if the PH assumption is not met; while the Cox PH and the promotion time models should not be used [43, 45]. Therefore, in the present study, despite the non-establishment of the PH assumption for TB, a mixture cure model was used.

Zhang presented a suitable alternative for modeling survival data with cure fraction and no proportional treatment effects as the accelerated hazard mixture cure model [46]. Another approach to non-proportional hazard alternatives is a lag-time model proposed by Zhang and Quan [47]. Also, Liu et al. suggested that generalized accelerated hazard cure models can be applied when the PH assumption is violated [48].

Beretta introduces a package that implements an extension of the semi-parametric proportional hazards cure model with time-varying covariates and a variable selection technique based on SCAD penalized likelihood [49].

In accordance with the above points, in future study, it is suggested the proposed models apply to these data to check whether there is a significant difference between the results of the present study or not.

We tried to follow both the mixture cure frailty model and semiparametric PH mixture cure model to check which model was better. For the mixture cure frailty model, the goodness of fit index (K-index) value was 0.65, but in the semiparametric PH mixture cure model, this value was equal to 0.62. So, this index showed that the mixture cure frailty model was better. Therefore, we reported the results based on the mixture cure frailty model. In the mixture cure models, the population was classified into two subpopulations composed: patients with long-term survival time, the probability of whom was estimated using a logistic model, and the patients with short-term survival that were estimated using a conventional Cox model. The applied mixture cure frailty model showed that the variables of gender, prison history, age, marital status, education, mode of HIV transmission, and ART were statistically significant in long-term survival time. Also, variables of prison history, mode of HIV transmission, ART, and tuberculosis affected the ​short-term survival time.

According to the results, the risk of death was higher for older ages, consistent with some studies and inconsistent with others [34–37]. Men were also at higher risk of death than women, consistent with other studies, probably because most people living with HIV were men [34, 36, 50]. In the present study, people who did not receive ART treatment had lower survival than those who received treatment, which was consistent with the results of other studies [51–53]. Many studies have also reported a significant positive association between HIV transmission through injecting drug use and death, which is in line with the present study [54, 55]. In the present study, in line with Rubaihayo’s study [50], people with lower levels of education were more at risk of death. Tabarsi et al. [56] examined the effect of different covariates on the mortality of people living with HIV/TB. They found that tuberculosis infection had a significant effect on death, consistent with the present study. Also, in their study, unlike the present work, no significant relationship existed between prison history and death. In the present study, in line with other studies, having a prison history reduced mortality and increased the lifetime of HIV patients [57]. As Dolan [58] noted in his study, healthcare facilities were provided for prisoners without any cost, which could be an opportunity to prevent and treat HIV. In addition, non-prisoners are more prone to high-risk behaviors such as injecting drug use, having abnormal sex, etc., which increase the risk of death. In the present study, in agreement with Kposowa [59], a significant association was found between marital status and the probability of death, as single individuals had a higher risk of death than married individuals.

The total number of variables was 10. Since the interest of health policymakers was to evaluate the effects of all variables on the long-term survival of HIV patients, we did not use any variable selection in this study, while Sun applied variable selection under a non-mixture-cure model for the prostate cancer [60].

One of the limitations of this study was due to using retrospectively collected data by health centers; it was not possible to examine the impact of other influential variables. Our suggestion for future work is to perform prospective cohort studies using the applied model in order to evaluate the survival of HIV patients. Moreover, a prospective study could be performed in Iran to examine the effect of genetic factors [61–63] and behavioral variables on the long- and short-term survival time of HIV patients.

## Conclusion

This study showed that the frailty mixture cure models were more appropriate in a situation where the study population was composed of two groups of susceptible and non-susceptible to the event of death. Also, using mixture cure models, the variables affecting short- and long-term survival time can be identified. The results of this study show that people with a prison history, people who received ART treatment, and people who contracted HIV through injection drug use survive longer. Health professionals should pay more attention to these findings in the field of HIV prevention and treatment.

## Supplementary Information

**Additional file 1.**

## Data Availability

The datasets analyzed during the current study are not publicly available due [Because the data is related to HIV and belongs to the Vice-Chancellor for Health of Kermanshah University of Medical Sciences, it must be with the permission of this Vice-Chancellor] but are available from the corresponding author on reasonable request.

## Abbreviations

TB: Tuberculosis
HIV: Human immunodeficiency virus
ART: Antiretroviral therapy
ELISA: Enzyme-linked immunosorbent assay
AIDS: Immunodeficiency syndrome
EM algorithm: Expectation–maximization algorithm
DIC: Deviance information criteria
LPML: Logarithm of pseudo-marginal likelihood
VIF: Variance inflation factor
