# Supplementary material for: The estimation of long and short term survival time and associated factors of HIV patients using mixture cure rate models
Source: BMC Med Res Methodol. 2023 May 22;23:123. doi: 10.1186/s12874-023-01949-x (PMC10201694; doi:10.1186/s12874-023-01949-x)
Supplement: Supplementary file 1 — Additional file 1. [file 12874_2023_1949_MOESM1_ESM.docx]

The probability of cure is calculated according to the following formula:

$$1-\hat{p}=1-(\exp(\mathbf{Z}\hat{\mathbf{\theta}})/(1+\exp(\mathbf{Z}\hat{\mathbf{\theta}})))$$

| **The possibility of a cure( unadjusted)** | **Censorship percentage** | **Variable** |
| --- | --- | --- |
| 0.37\|0.46 | 57.9\|57.0 | Age(>33\|<=33) |
| 0.38\|0.74 | 51.5\|87.7 | Gender (Male\| Female) |
| 0.49\|0.36 | 66.8\|47.3 | Marital Status (Married\| Single) |
| 0.75\|0.23 | 86.0\|33.4 | Antiretroviral therapy (Yes \|No) |
| 0.51\|0.26 | 66.2\|39.0 | Tuberculosis infection (Yes\| No) |
| 0.71\|0.41 | 84.4\|56.6 | Education (High \|Low) |
| 0.44\|0.37 | 56.3\|59.4 | Imprisonment(Yes \|No) |
| 0.40\|0.49 | 52.5\|71.8 | Drug Abuse(Yes \|No) |
| 0.37\|0.46 | 51.8\|61.6 | Occupational status (Employed\| Unemployed) |
| 0.36\|0.61 | 45.1\|82.8 | Modes of HIV transmission (Injecting drug users\| Other) |

**Table S1.** Estimation unadjusted probability of cure.

| 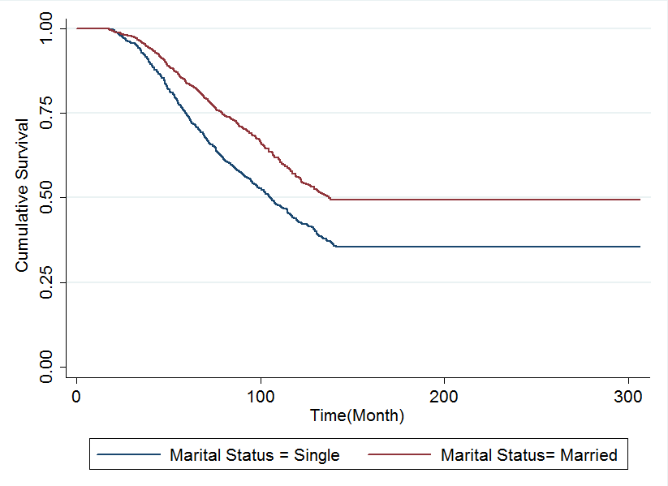  (b) | 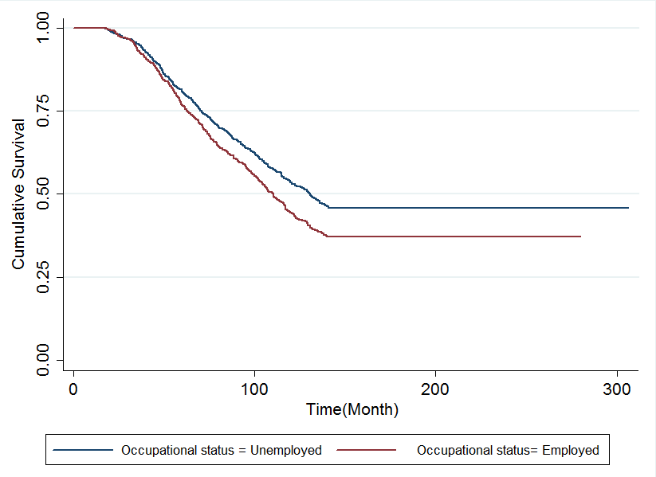  (a) |
| --- | --- |
| 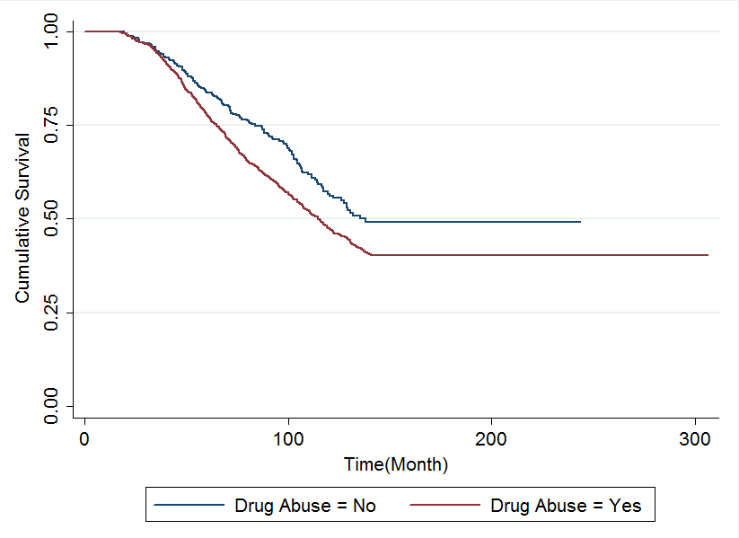  (d) | 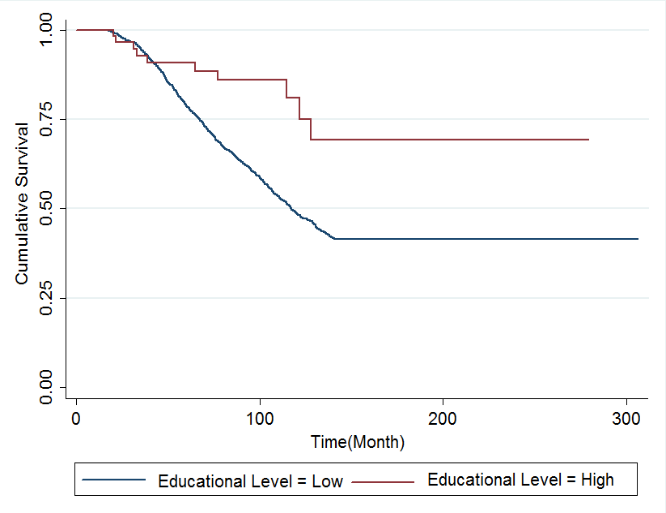  (c) |
| 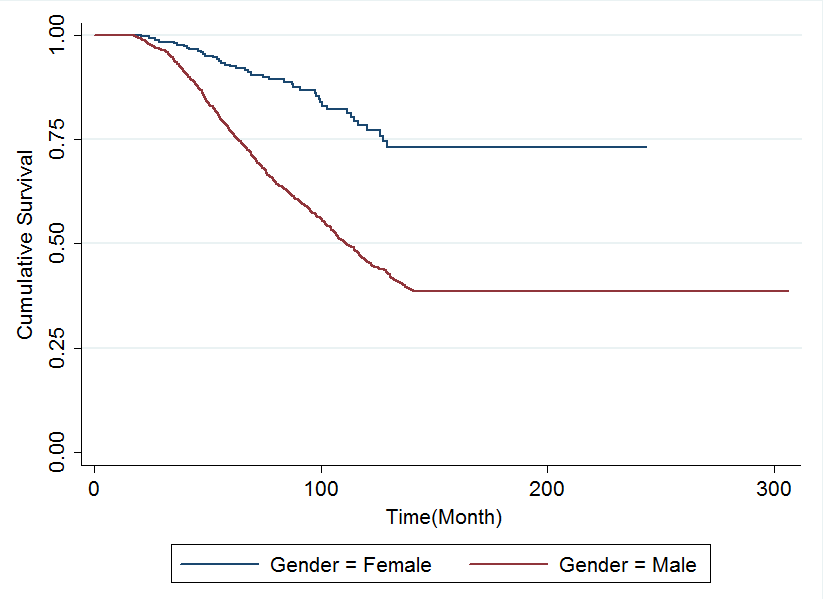  (f) | 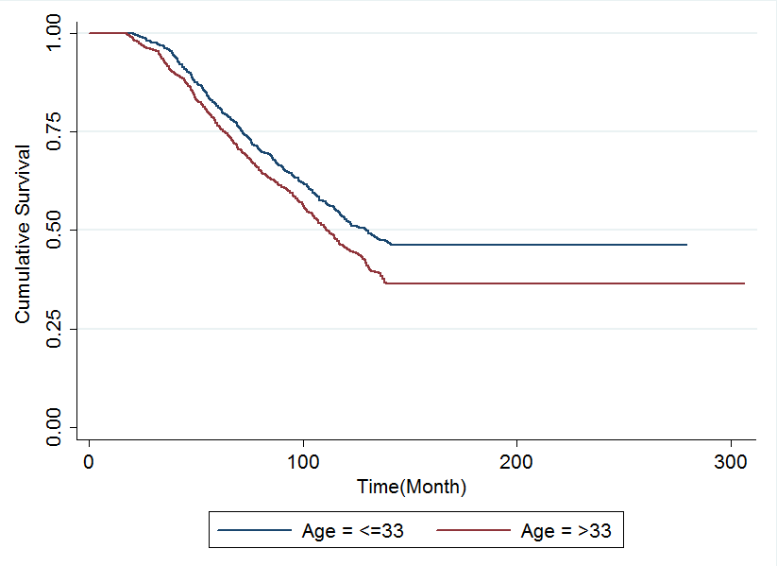  (e) |
| 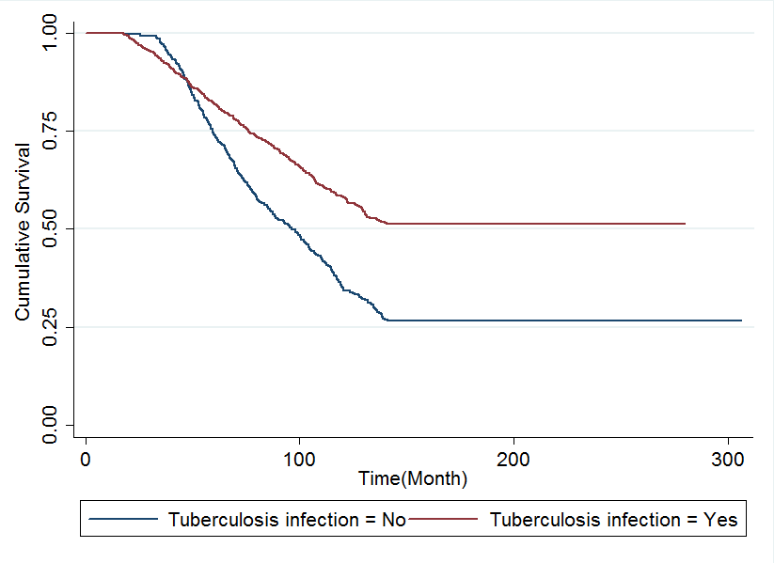  (g) | |

**Figure S1.** (a) Kaplan-Meier survival curves for occupational status; (b) Kaplan-Meier survival curves for marital status; (c) Kaplan-Meier survival curves for education; (d) Kaplan-Meier survival curves for drug abuse: (e) Kaplan-Meier survival curves for age; (f) Kaplan-Meier survival curves for gender; (g) Kaplan-Meier survival curves for tuberculosis infection
